# Endoscopy Guided Eustachian Tube Balloon Dilation: Our Experiences

**DOI:** 10.22038/ijorl.2019.41623.2359

**Published:** 2020-09

**Authors:** Santosh-Kumar Swain, Sunil Janardan, Jatindra-Nath Mohanty

**Affiliations:** 1 *Department of Otorhinolaryngology, IMS and SUM Hospital, Siksha “O” Anusandhan University (Deemed to be), K8, Kalinga Nagar, Bhubaneswar-751003, Odisha, India.*; 2 *Kerala ENT Research Foundation, Kollam, Kerala, India.*; 3 *Medical Research Laboratory, IMS and SUM Hospital, Siksha “O” Anusandhan University (Deemed to be), K8, Kalinga Nagar, Bhubaneswar-751003, Odisha, India.*

**Keywords:** Balloon dilatation, Computed tomography, Eustachian tube, Eustachian tube dysfunction

## Abstract

**Introduction:**

Eustachian tube (ET) dysfunction is a common clinical entity but its treatment is still challenging to Otorhinolaryngologists. This study is done to know the effectiveness of transnasal endoscopic balloon dilatation of eustachian tube for treatment of chronic eustachian tube dysfunction.

**Materials and Methods:**

It is a retrospective observational study conducted between May 2018 to June 2019 at IMS and SUM Hospital, Siksha 'O' Anusandhan University, Bhubaneswar, Odisha, India. Twenty one patients were identified with diagnosis of ET dysfunction and assigned to this study. The transnasal endoscopic procedure was done to dilate the cartilaginous part of the eustachian tube with a balloon catheter. Preoperative computed tomography was done in all cases. All patients were post-operatively assessed in 1^st^, 2^nd^ and 8^th^ weeks after the procedure.

**Result:**

Balloon dilatation of the eustachian tube was easily performed in all cases of this study. No abnormality including carotid canal was seen before this procedure. All except 2 cases revealed significant improvement in the ET functions. There was no damage to any vital structures like internal carotid artery in this study.

**Conclusion:**

The majority of the patients participated in this study showed positive outcome after balloon dilation of eustachian tube. It is a feasible and safe procedure for dilating the eustachian tube. This treatment is a very promising and requires more research on this aspect.

## Introduction

The evolution of rigid endoscope for endoscopic sinus surgery along with inflatable balloons for sinus surgery opened up new possibilities for treatment of eustachian tube (ET) dysfunction. Progressive development of flexible fiberoptic endoscopy has made hope for the first time to perform an atraumatic endoscopy of the ET (1).The anatomical and physiological description of the eustachian tube was first done by Italian physician BartolomeusEustachius in 1562, which dramatically changed the concept of this structure and etiopathology of middle ear diseases (2).

Eustachian tube dysfunction is a common clinical entityencounteredbyotolaryngologists. ET dysfunction is usually seen in at least 1% of the adult population (3).

The clinical presentations of the Eustachian tube dysfunctions are fullness of the ear, decreased hearing, otalgia, tinnitus and vertigo. The medical treatments of ET dysfunction are antihistamines, nasal decongestants and oral or nasal steroids.If conservative treatment for ET dysfunction fails, eustachiantuboplasty may be done with help of microdebrider or laser to remove hypertrophic mucosa and cartilage on the nasopharyngeal opening of the eustachian tube. Currently eustachian tube balloon dilation is a new surgical technique which should be verified for its *efficacy*. The aim of this study is to assess the indications, surgical technique, complications and effectiveness of the endoscopy guided transnasal balloon dilation of the eustachian tube.

## Materials and Methods

It is a retrospective observational study and this study was done between May2018 to June 2019 at IMS and SUM Hospital, Siksha 'O' Anusandhan University, Bhubaneswar, Odisha, India. This study was approved by institutional ethics committee (IEC) with reference number of IMS/CRL/IEC/22/02.03. 2018.

Informed consents were taken from all of the patients those participated in this study. There were 21 patients underwent transnasal endoscopic balloon dilatation of the eustachian tube under general anesthesia for chronic eustachian tube dysfunction. Out of the 21 patients, there were 14 male patients and 7 female patients in this study. The inclusion criteria for this study were ET dysfunction for more than 3 months, significant symptoms during flying, recurrent serous otitis media in a year and age more than 18 years (adult patients). 

Symptoms of poor ET functions were confirmed like feeling of ear block, unilateral or bilateral partial hearing loss, clicking noises or tinnitus and vertigo, which are reversible or not after Valsalva maneuver, swallowing or yawning. 

The exclusion criteria for participating in this study were known pathology at the nasopharynx such as growth or anatomical abnormalities like cleft palate, large polyp at the nasopharynx near nasopharyngeal opening of the ET, gross septal deviation, hypertrophied turbinates, absence of bony covering of the internal carotid artery (findings from pre-operative computed tomography (CT) scan), abnormalities in palate, age under the 18 years old and patients unfit for general anesthesia. 

All patients preoperatively underwent detail clinical examinations, pure tone audiometry, tympanometry and a thin layer CT scan of the temporal bone/petrosal bone of both sides. All the patients underwent standard endoscopic examinations of the nose and nasopharynx to rule out any pathology in the nose and nasopharynx. All patients also underwent microscopic examinations of the ears. Pre-operative and post-operative assessment of clinical symptoms, Valsalva maneuver, tympanometry and post-operative complications were analyzed.

**Surgical procedure**

This procedure was done under general anesthesia with topical application of nasal decongestants into nasal cavity and nasopharynx. This procedure was offered after subjective, physical and Otorhinolaryn- gological examinations. 

**Fig1:**
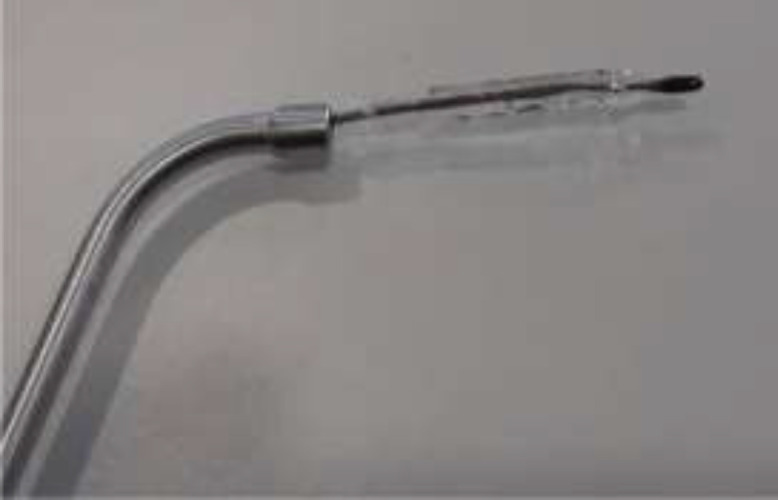
Balloon catheter for eustachian tube dilation

**Fig2:**
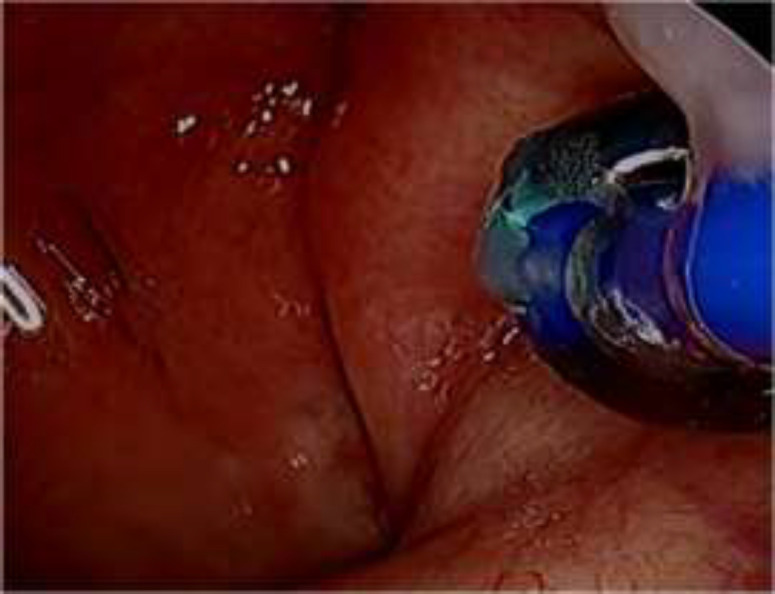
Balloon catheter is pushed into the cartilaginous part of the Eustachian tube

**Fig3:**
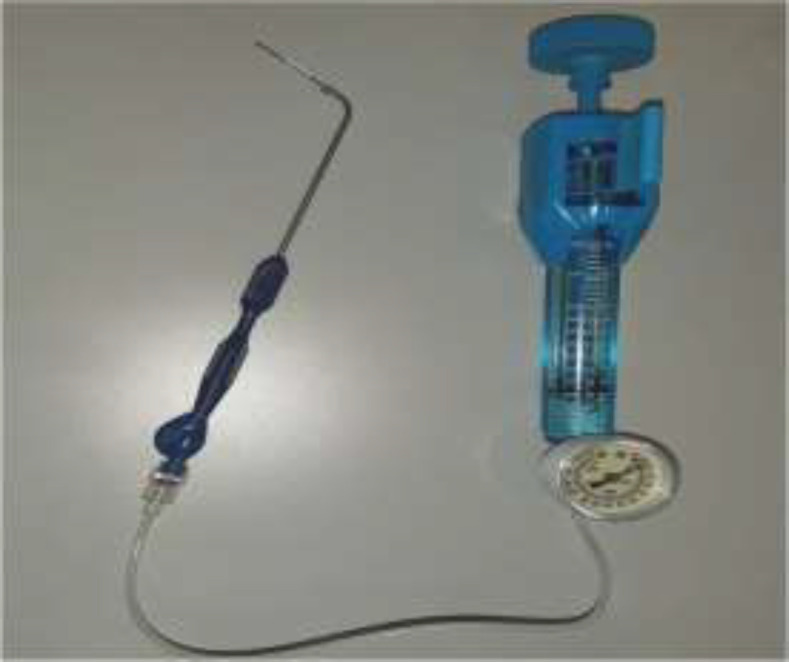
Pressure device (Inflation pump) which is attached to the balloon catheter

A specially designed 500µm diameter balloon catheter(Spiggle and Theis Company, Overath, Germany) (Fig.1) was introduced into the eustachian tube for a length of 20mm via a special insertion instrument under guidance of rigid nasal endoscope. The catheter was inserted into nasopharyngeal orifice of the ET (Fig.2). The balloon was inflated by sterile water with a pressure of ten bars for two minutes by help of pressure device or inflation pump (Fig.3).

The balloon was emptied and removed. The aim was to dilate the cartilaginous part of the eustachian tube without any structural damage. The successful technique is always associated with dilatation of cartilaginous part of the ET along with symptomatic improvement of the patient without any intra-operative and post-operative complications. This is a day care procedure where patient admitted in the morning and discharged in afternoon. After day of the procedure, all the patients were reviewed at outpatient department for checking any complications and were advised to do Valsalva maneuver three times a day for two weeks. Two nasal sprays were used after the procedure like nasal decongestants for 7days and corticosteroids for 2 weeks. The nasal spray was given to clear any nasal allergy in post-operative period, so that it will not interfere with positive outcome due to balloon dilatation of ET. All the patients were underwent review check up at the outpatient department on 1^st^ and 2^nd^ week of procedure. 

**Fig 4:**
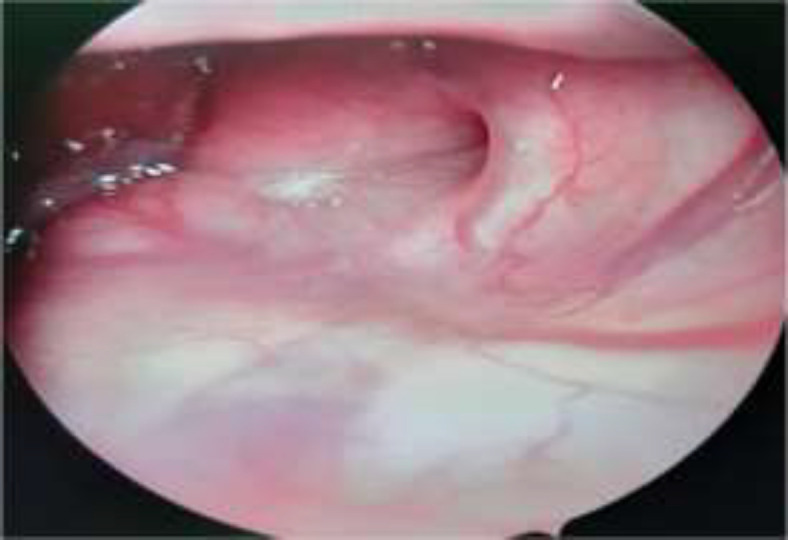
The endoscopic picture of the pharyngeal orifice of the Eustachian tube after 2months of balloon dilation

After 2 months of procedure, the patients were reviewed for audiological tests again. All the patients were asked questionnaire after two months of procedure on the basis of overall improvement of symptoms with the procedure. Nasopharyngeal opening of the ET was also assessed after 2 months of surgery to find out its patency (Fig.4).

## Results

There were total 21 patients participated in this study. Out of 21 patients, 14 were males and 7 females. In 4 patients procedures were done in both sides. The mean age of the patients was 44.90 years and age ranged from 19 to 64 years. Out of 21 patients, 14 patients had chronic persistent ET dysfunctions, 5 patients had intermittent ET dysfunctions and 2 patients had recurrent glue ear (Table.1).

**Table1:** Profile of patients those undergoing Balloon dilation of eustachian tube

**Patient**	**Age in years**	**Gender**	**Indications**	**Investigations**	**Sides of ET**	**Complications**
1	19	Male	Intermittent ETD	PTA,Tymp,CT scan	Right side	Nil
2	21	Male	Persistent ETD	PTA,Tymp,CT scan	Right side	Nil
3	25	Male	Persistent ETD	PTA,Tymp,CT scan	Left side	Mild epistaxis
4	29	Female	Persistent ETD	PTA,Tymp,CT scan	Right side	Nil
5	35	Male	Intermittent ETD	PTA,Tymp,CT scan	Right side	Nil
6	35	Female	Persistent ETD	PTA,Tymp,CT scan	Left side	Nil
7	39	Male	Recurrent Glue ear	PTA,Tymp,CT scan	Right side	Acute otitis media
8	41	Male	Persistent ETD	PTA,Tymp,CT scan	Left side	Nil
9	43	Female	Intermittent ETD	PTA,Tymp,CT scan	Bilateral	Nil
10	44	Male	Persistent ETD	PTA,Tymp,CT scan	Right side	Mild epistaxis
11	45	Male	Persistent ETD	PTA,Tymp,CT scan	Bilateral	Nil
12	49	Male	Persistent ETD	PTA,Tymp,CT scan	Left side	Nil
13	52	Female	Intermittent ETD	PTA,Tymp,CT scan	Left side	Nil
14	53	Female	Persistent ETD	PTA,Tymp,CT scan	Left side	Nil
15	55	Male	Persistent ETD	PTA,Tymp,CT scan	Right side	Nil
16	56	Male	Recurrent Glue ear	PTA,Tymp,CT scan	Right side	Nil
17	57	Male	Intermittent ETD	PTA,Tymp,CT scan	Bilateral	Nil
18	58	Female	Persistent ETD	PTA,Tymp,CT scan	Right side	Nil
19	61	Male	Persistent ETD	PTA, Tymp,CT scan	Left side	Nil
20	62	Male	Persistent ETD	PTA, Tymp, CT scan	Left side	Nil
21	64	Female	Persistent ETD	PTA, Tymp, CT scan	Right side	Nil

ETD: Eustachian dysfunction, PTA: Pure tone audiometry, Tymp: Tympanometry, CT: Computed tomography scan.

There were 15 patients showed type-C tympanogram 6 had type-B tympanogram before procedure. Out of 21 patients, 3 were underwent tympanostomy tube in past but failed to resolve the ET dysfunction. Here, 18 patients underwent unilateral ET balloon dilation whereas 3 patients underwent bilateral ET balloon dilation. Out of 18 unilateral ET balloon dilations, 10 patients underwent in right side and 8 patients in left sides. After procedure, there were type-A tympanogram in 17 patients and no change in 4 patients. Otoscopy before and after procedure were compared. In all patients, tympanic membranes were not normal before procedure and 19 cases becomes normal tympanic membrane after surgery (2 months after procedure) except 2 cases (Table.2).

**Table2:** Pre-operative and post-operative findings in patients undergoing Balloon dilation of ET

**Patient**	**Pre-operative Otoscopy**	**Post-operative** **Otoscopy**	**Pre-operative** **Valsalva**	**Post-operative Valsalva**	**Pre-operative** **Tympanometry**	**Postoperative tympanometry**
1	TM retraction	Normal	-Ve	+Ve	Type-C	Type-A
2	TM retraction	Normal	-Ve	+Ve	Type-C	Type-A
3	TM looks dull	Normal	-Ve	+Ve	Type-C	Type-A
4	TM retraction	Normal	-Ve	-Ve	Type-C	Type-A
5	Air-fluid level behind TM	Normal	-Ve	+Ve	Type-B	No change
6	TM retraction	Normal	-Ve	+Ve	Type-C	Type-A
7	TM retraction	Retraction persists	-Ve	+Ve	Type-C	Type-A
8	TM retraction	Normal	-Ve	+Ve	Type-C	No change
9	Air-fluid level behind TM	Normal	-Ve	-Ve	Type-B	Type-A
10	TM retracted	Normal	-Ve	+Ve	Type-C	Type-A
11	TM retracted	Normal	-Ve	+Ve	Type-C	Type-A
12	Dull TM	Normal	-Ve	+Ve	Type-B	No Change
13	Dull TM	Normal	-Ve	+Ve	Type-B	Type-A
14	Retracted TM	Normal	-Ve	+Ve	Type-C	Type-A
15	Retracted TM	Normal	-Ve	+Ve	Type-C	Type-A
16	Dull TM	Normal	-Ve	+Ve	Type-B	Type-A
17	Dull TM	Normal	-Ve	-Ve	Type-B	Type-A
18	Retracted TM	Persists TM retraction	-Ve	+Ve	Type-C	Type-A
19	Retracted TM	Normal	-Ve	+Ve	Type-C	No Change
20	Retracted TM	Normal	-Ve	+Ve	Type-C	Type-A
21	Retracted TM	Normal	-Ve	+Ve	Type-C	Type-A

Valsalva maneuver was done in all cases before and after procedure. All cases showed positive (normal/patent ET) Valsalva except 3 cases after balloon dilation of eustachian tube. Before ET dilatation, CT scan was done in all cases which did not show any significant abnormalities. 

There was no evidence of carotid artery aneurysm, tumors or malformations in the course of eustachian tube. Postoperative CT scan were also done in all cases which showed no change in bony lumen of the eustachian tube. There were no fractures seen in bony part of the eustachian tube. During procedures, two patients presented minor bleeding from eustachian opening whereas rest were uneventful. One patient developed acute otitis media after 48 hours of the procedure for which patient treated with medications like antibiotics, antihistamines and nasal decongestants. All patients improved symptomatically except two cases.

## Discussion

The eustachian tube (ET) is an uncommon location for intervention because of its anatomical site, uncertain function and anecdotal reports for catastrophic situations like rupture of internal carotid artery following inadvertent injection of Teflon (4). 

The approximate length of eustachian tube in adults is 37.5mm long and has bony and cartilaginous parts, extending from nasopharynx to the middle ear (5). The physiological functions of the eustachian tube are pressure regulation of the middle ear, ventilation, drainage of the middle ear secretions, protection of the middle ear from nasopharyngeal secretions and sound pressure (6). There are current research utilizing video endoscopy helping to improve the understanding of eustachian tube functions and its role in middle ear pathology, identifying the common site for dysfunction at cartilaginous part (7). 

Dysfunction of the eustachian tube is defined as failure of the functional valve of the eustachian tube to open and/or close properly. The etiopathogenesis of the ET dysfunction are allergic rhinitis, laryngopharyngeal reflux, mechanical obstruction by nasopharyngeal growth, primary ciliary dyskinesia and neuromuscular dysfunction. A new technique of rigid nasal endoscopy for dilating eustachiantube was developed by Yamashita in 1983 by the use of the flexible fiberscope with an instrument channel to insufflate air for expanding the lumen of the eustachian tube (8). Eustachian dysfunction is a disorder which results in inadequate middle ear ventilation, leads to aural fullness, hearing loss and tinnitus. It may complicate into serous otitis media, retraction of tympanic membrane and cholesteatoma formation (9).ET dysfunction affects approximately 1 per 100 adults (10,11). Eustachian tube dysfunction is a physiological disorder which may be temporary and spontaneously resolving. Eustachian tube dysfunction lasting for over 3 months is usually called as chronic eustachian dysfunction. It is usually a poorly defined clinical condition with variable diagnostic criteria on the basis of clinical presentations, otoscopic findings and tympanometry results (10). 

Its debilitating symptoms often affect quality of life due to persistent sensation of fullness in the ear, otalgia and uncomfortable air travel or scuba diving. Prolonged ET dysfunction may cause conductive hearing loss and cholesteatoma formation. On examination, there may be serous otitis media or negative middle ear pressure (in tympanometry). Sequel of eustachian tube dysfunctions are retraction pockets, perforation, chronic otorrhea and cholesteatoma.The routine investigations done before balloon dilation of ET are pure tone audiometry, tympanometry and CT scan of the bilateral temporal bone. These tests are performed in all cases those will undergo balloon dilation of the ET. Functional MRI (Magnetic resonance imaging) is useful to differentiate from mucosal obstruction from structural obstruction (12). 

Pure tone audiometry assesses the hearing loss whereas tympanometry will assess the middle ear status. CT scan will assess the status of bony part of ET and canal for internal carotid artery. 

There was little evidence of effectiveness for present medical and surgical interventions for EDT like nasal decongestants, systemic and topical corticosteroids, antihistamines, mechanical devices and nasal/ septal surgery (10). One study showed that no improvement of ETD after six weeks treatment of topical nasal steroids (13). The surgical treatment for ET dysfunction is myringotomy and grommet placement in the tympanic membrane which helps to equalize the middle ear pressure and drainage of the middle ear fluid via grommet. It effectively bypasses the ET and relieves the symptoms but does not cure the Eustachian tube dysfunction. Grommet or tympanostomy tubes often require frequent replacement multiple times if ET dysfunction persists for longer period. Tympanostomy tubes can damage the tympanic membrane, persistent perforation, infection and extrusion. This makes a financial burden on patient’s family which adds to patient inconvenience and discomfort.

Current conventional treatment for ET dysfunction may not be effective always. The target for balloon dilatation of ET is 8 to 12 mm cartilaginous segment of ET which acts as a valve within cartilaginous part of the ET where physiological deficiency seen in chronic ET dysfunction (14). 

**Fig 5:**
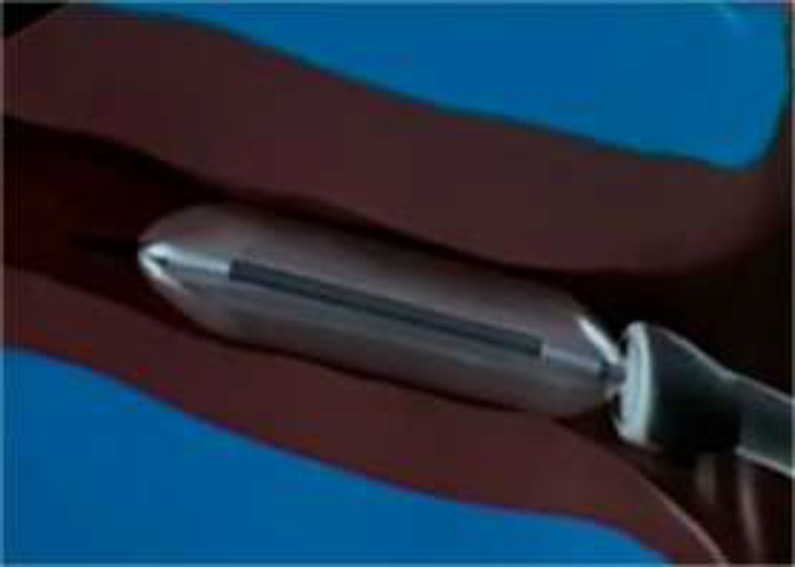
Cartilaginous part of the Eustachian tube during balloon dilation

This is done under transnasal endoscopic vision. So optimum care should be taken not to push the balloon catheter past the cartilaginous part of the ET and bony isthmus or to use large size balloon which too large for the patient. Balloon eustachian tuboplasty is less invasive in comparison to laser or microdebrider Eustachian tuboplasty. The mechanisms for eustachian tube balloon dilation include both widening of the cartilaginous part of the ET and histopathological changes (15) (Fig.5). 

A study on histopathological changes after balloon dilatation of ET revealed the crushing effect of balloon on inflammatory cells on the mucosal lining of the ET lumen with sparing of the basal layer and rapidly replacing the inflamed mucosal lining with a fibrous scar (15). Histopathological analysis showed decreased inflammation in the epithelial surface and submucus tissues. So, there is net reduction of the inflammation in the lumen of ET and improvement in clinical functions of the eustachian tube after surgery (16). 

There are certain theoretical complications like internal carotid artery rupture, permanent conductive hearing loss, damage to the ET, scarring, stenosis, middle ear infections and pain (17). 

Eustachian tubal dilatation is usually avoided in pediatric age group. One study reported minor epistaxis in balloon dilatation (18). There were serious complications like subcutaneous emphysema reported in another study (19). Acute otitis media was also reported as complication of balloon dilatation of ET, for which post-operative prophylactic antibiotics are advised (19). The proximity of the internal carotid artery has clinical importance as often require avoiding catastrophic injuries and death which has been anecdotally documented in the past during injection procedures in patulous ET (20). 

There were no cases reported for complications of carotid artery injury or patulous ET. But there is theoretical risk for injury to a dehiscent carotid artery which runs adjacent to the bony part of the ET (21). CT scan of the petrous temporal bone is required as part of the pre-operative investigations. One study conducted a retrospective analysis of petrous temporal bone CT scan those underwent ET dilatation where authors found 24 balloon dilatations were done in 17 patients with carotid artery canal dehiscence with no complications or any difficulties (22).

It suggests that routine CT scan of the petrous temporal bone before eustachian dilatation is not indicated. In this study, there was lack of mucosal injury in lumen of the ET to avoid post-dilatation strictures. Balloon eustachian tubal dilatation is a minimal invasive technique where risks are often minimized. 

The inflatable balloon help to deliver maximum 19.88 mmHg pressure per mm^2^ surface area in the lumen of the ET which may give pressure on the mucosal capillaries in this area. This may lead to localized and temporary edema but it should be avoided for marked mucosal ischemia and necrosis with formation of scar. In this study, endoscopic guided transnasal balloon dilation of ET has been found to be feasible and safe to inflate the ET and significantly help to improve the ET functions as in other study (23).

Although there is no such gold standard method for eustachian tube function, the outcomes or benefits of this surgery are observed on the basis of four factors: Tympanometry profile (Normal-Type-A, Abnormal-Type-B and Type-C), Otoscopic pictures (Normal or abnormal) (retracted tympanic membrane), Patient symptoms (Improved, not improved or worsened) and Valsalva (Always positive, occasional positive or negative).

Outcomes of endoscopic guided transnasal balloon dilation of ET are divided into short term(≤ 6months) and long term(≥ 6months). Long-term studies are required to evaluate properly the long lasting benefits and safety of balloon ET dilatation in the treatment of the chronic eustachian tube dysfunction. Now ET balloon dilation is emerged as a surgical option for Eustachian tube dysfunction which targets the cartilaginous part of the ET.

## Conclusion

Endoscopy guided balloon dilatation of eustachian tube is a novel technique to perform minimal invasive eustachian tube dilation for improvement of the ET dysfunction. The objective of the balloon eustachiantuboplasty is to widen the cartilaginous part of the eustachian tube and enhances the physiological functions with minimal or no complications. This minimally invasive technique for ET dysfunction is proved to be feasible and safe in the treatment of the ET dysfunction. This is a low risk surgical procedure where postsurgical pain is minimal and patients can return to normal activities following a day.
